# Boolean Modeling Reveals the Necessity of Transcriptional Regulation for Bistability in PC12 Cell Differentiation

**DOI:** 10.3389/fgene.2016.00044

**Published:** 2016-04-14

**Authors:** Barbara Offermann, Steffen Knauer, Amit Singh, María L. Fernández-Cachón, Martin Klose, Silke Kowar, Hauke Busch, Melanie Boerries

**Affiliations:** ^1^Systems Biology of the Cellular Microenvironment Group, Institute of Molecular Medicine and Cell Research, Albert-Ludwigs-University FreiburgFreiburg, Germany; ^2^German Cancer ConsortiumFreiburg, Germany; ^3^German Cancer Research CenterHeidelberg, Germany

**Keywords:** PC12 cells, Boolean modeling, NGF signaling, EGF signaling, bistability

## Abstract

The nerve growth factor NGF has been shown to cause cell fate decisions toward either differentiation or proliferation depending on the relative activity of downstream pERK, pAKT, or pJNK signaling. However, how these protein signals are translated into and fed back from transcriptional activity to complete cellular differentiation over a time span of hours to days is still an open question. Comparing the time-resolved transcriptome response of NGF- or EGF-stimulated PC12 cells over 24 h in combination with protein and phenotype data we inferred a dynamic Boolean model capturing the temporal sequence of protein signaling, transcriptional response and subsequent autocrine feedback. Network topology was optimized by fitting the model to time-resolved transcriptome data under MEK, PI3K, or JNK inhibition. The integrated model confirmed the parallel use of MAPK/ERK, PI3K/AKT, and JNK/JUN for PC12 cell differentiation. Redundancy of cell signaling is demonstrated from the inhibition of the different MAPK pathways. As suggested *in silico* and confirmed *in vitro*, differentiation was substantially suppressed under JNK inhibition, yet delayed only under MEK/ERK inhibition. Most importantly, we found that positive transcriptional feedback induces bistability in the cell fate switch. *De novo* gene expression was necessary to activate autocrine feedback that caused Urokinase-Type Plasminogen Activator (uPA) Receptor signaling to perpetuate the MAPK activity, finally resulting in the expression of late, differentiation related genes. Thus, the cellular decision toward differentiation depends on the establishment of a transcriptome-induced positive feedback between protein signaling and gene expression thereby constituting a robust control between proliferation and differentiation.

## 1. Introduction

The rat pheochromocytoma cells PC12 are a long established *in vitro* model to study neuronal differentiation, proliferation and survival (Greene and Tischler, 1976; Burstein et al., 1982; Cowley et al., 1994). After stimulation with the nerve growth factor (NGF), a small, secreted protein from the neurotrophin family, PC12 cells differentiate into sympathetic neuron-like cells, which is morphologically marked by neurite outgrowth over a time course of up to 6 days (Levi-Montalcini, 1987; Chao, 1992; Fiore et al., 2009; Weber et al., 2013). NGF binds with high affinity to the TrkA receptor (tyrosine kinase receptor A), thereby activating several downstream protein signaling pathways including primarily the protein kinase C/phospholipase C (PKC/PLC), the phosphoinositide 3-kinase/protein kinase B (PI3K/AKT) and the mitogen-activated protein kinase/extracellular signal-regulated kinase (MAPK/ERK) pathways (Kaplan et al., 1991; Jing et al., 1992; Vaudry et al., 2002). Beyond these immediate downstream pathways, further studies showed the involvement of Interleukin 6 (IL6), Urokinase plasminogen activator (uPA) and Tumor Necrosis Factor Receptor Superfamily Member 12A (TNFRSF12A) in PC12 cell differentiation (Marshall, 1995; Wu and Bradshaw, 1996; Leppä et al., 1998; Xing et al., 1998; Farias-Eisner et al., 2000, 2001; Vaudry et al., 2002; Tanabe et al., 2003). Sustained ERK activation is seen as necessary and sufficient for the successful PC12 cell differentiation under NGF stimulation (Avraham and Yarden, 2011; Chen et al., 2012), whereas transient ERK activation upon epidermal growth factor (EGF) stimulation results in proliferation (Gotoh et al., 1990; Qui and Green, 1992; Marshall, 1995; Vaudry et al., 2002). In fact, selective pathway inhibition or other external stimuli that modulate the duration of ERK activation likewise determine the cellular decision between proliferation and differentiation (Dikic et al., 1994; Vaudry et al., 2002; Santos et al., 2007). Consequently, the MAPK signaling network, as the key pathway in the cellular response, has been studied thoroughly *in vitro* and *in silico* (Sasagawa et al., 2005; von Kriegsheim et al., 2009; Saito et al., 2013). Interestingly, both EGF and NGF provoke a similar transcriptional program within the first hour. Therefore, differences in cellular signaling must be due (i) to differential regulation of multiple downstream pathways and (ii) late gene response programs (>1 h) that feed back into the protein signaling cascade. As an example for pathway crosstalk, both, the MAPK/ERK and c-Jun N-terminal kinase (JNK) pathways regulate c-Jun activity and are necessary for PC12 cell differentiation (Leppä et al., 1998; Waetzig and Herdegen, 2003; Marek et al., 2004), while uPA receptor (uPAR) signaling, as a result of transcriptional AP1 (Activator Protein-1) regulation, is necessary for differentiation of unprimed PC12 cells (Farias-Eisner et al., 2000; Mullenbrock et al., 2011).

In the present study, we combined time-resolved transcriptome analysis of EGF and NGF stimulated PC12 cells up to 24 h with inhibition of MAPK/ERK, JNK/JUN, and PI3K/AKT signaling, to develop a Boolean Model of PC12 cell differentiation that combines protein signaling, gene regulation and autocrine feedback. The Boolean approach allows to derive important predictions without detailed quantitative kinetic data and parameters over different time scales (Singh et al., 2012). Protein signaling comprised MAPK/ERK, JNK/JUN, and PI3K/AKT pathways. Based on the upstream transcription factor analysis and transcriptional regulation of *Mmp10* (Matrix Metallopeptidase 10), *Serpine1* (Serpin Peptidase Inhibitor, Clade E, Member) and *Itga1* (Integrin, Alpha 1), we further included an autocrine feedback via uPAR signaling. The model topology was trained on the transcriptional response after pathway inhibition. Inhibition of JNK completely blocked PC12 cell differentiation and long-term expression of target transcription factors (TFs), such as various Kruppel-like factors (*Klf2, 4, 6* and *10*), *Maff* (V-Maf Avian Musculoaponeurotic Fibrosarcoma Oncogene Homolog F) and AP1. Interestingly, inhibition of MEK (mitogen-activated protein kinase kinase), blocking the phosphorylation of ERK, slowed down, but not completely abolished cell differentiation. Neurite quantification over 6 days confirmed a late and reduced, but significant PC12 differentiation, which hinted at alternative pathway usage through JNK. Inhibition of the PI3K/AKT pathway, which is involved in cell proliferation (Chen et al., 2012), even increased the neuronal morphology and neurite outgrowth.

In conclusion, our Boolean modeling approach shows the complex interplay of protein signaling, transcription factor activity and gene regulatory feedback in the decision and perpetuation of PC12 cell differentiation after NGF stimulation.

## 2. Materials and methods

### 2.1. Cell culture and stimulation

PC12 cells were obtained from ATCC (American Type Culture Collection, UK) and were cultured at 37°C at 5% CO_2_ in RPMI 1640 medium, supplemented with 10% Horse Serum, 5% Fetal Bovine Serum, 1% penicillin/streptomycin (PAN Biotech, Germany) and 1% glutamine (PAN Biotech, Germany). For cell stimulation, 500,000 cells/well were seeded on collagen coated 6 well plates (Corning, NY, USA). The following day, cells were stimulated with 50 ng/ml rat nerve growth factor (NGF; Promega, Madison, WI, USA) or 75 ng/ml epidermal growth factor (EGF; R&D Systems; Wiesbaden, Germany) for the corresponding times. For the pathway inhibition experiments, the following inhibitors were used and added 60 min before NGF was added, mitogen-activated protein inhibitor at a concentration of 20 μM (MEKi; U0126 from Promega, Madison, WI, USA), phosphoinositide 3-kinase inhibitor at a concentration of 40 μM (PI3Ki; LY-294002 from Enzo Life Sciences, New York, USA) and c-Jun N-terminal kinase inhibitor at a concentration of 20 μM (JNKi; SP600125 from Sigma-Aldrich, St. Louis, USA). The inhibitors were dissolved in DMSO and were further diluted in cell culture medium at their working concentration. Control cells were treated with DMSO at the same concentration that was present in the cells with inhibitor treatment.

### 2.2. RNA isolation and quantitative real time PCR (qRT-PCR)

Total RNA was isolated from 500,000 cells per timepoint according to the manufacturer's protocol (Universal RNA Purification Kit, Roboklon, Germany). RNA integrity was measured using an Agilent Bioanalyzer-2000 (Agilent Technologies GmbH, Waldbronn, Germany), and its content quantified by NanoDrop ND-1000 (Thermo Fisher Scientific, Wilmington, USA). For RT-qPCR, double strand cDNA was synthesized from 1 μg of total RNA using the iScript™ cDNA Synthesis kit (Quanta Biosciences, Gaithersburg, USA) according to the manufacturer instructions. RT-qPCR was performed in a CFX96 instrument (BioRad, Hercules, CA, USA) using a SYBR Green master mix. Relative gene expression levels were calculated with the 2-ΔΔCt method, using HPRT1 and 18S ribosomal RNA as reference genes. Post-run analyses were performed using Bio-Rad CFX Manager version 2.0 and the threshold cycles (Cts) were calculated from a baseline subtracted curve fit. See Supplementary Table 1 for primer pair sequences.

### 2.3. Microscopy and quantification

Live phase contrast images from PC12 cells under the different conditions were acquired using a Nikon Eclipse Ti Inverted Microscope (Nikon; Düsseldorf, Germany) equipped with a Perfect Focus System (PFS) and a Digital cooled Sight Camera (DS-QiMc; Nikon, Germany) as described in (Weber et al., 2013). Briefly, PC12 cells were cultured in collagen coated 6-well plates (500,000 cells/well) and treated as described in “Cell culture and stimulation” and 150 images per well, every second day were recorded with the same spatial pattern. Cell differentiation is calculated by the ratio of the two described imaging features (Weber et al., 2013) convex hull (CH) to cell area (CA) for 150 images per well over 6 days (Weber et al., unpublished data).

### 2.4. Western blot

For each timepoint and condition 3 × 10^6^ PC12 cells (for inhibition experiments) or 5 × 10^6^ PC12 cells (for EGF vs. NGF comparison) were seeded in 10cm collagen coated Cell BIND dishes (Corning; Germany). Cells were collected after 5, 10, 30 min, 1, 2, 4, 6, 8, 12, 24, and 48 h in 200 μl RIPA buffer (containing 0.5% SDS), supplemented with proteinase inhibitor (complete mini EDTA free tablets, Roche, Basel, Switzerland) and Benzonase (Merck), and lysed for 20 min under agitation. A total of 30 μg protein was loaded per lane and run in 10% SDS- polyacrylamide gels, transferred to polyvinylidene difluoride membranes. Membranes were cut horizontally into fragments according to the expected sizes of the protein of interest and immunoblotted with antibodies against total p44/42 (ERK1/2, 1:2000, #9102S, Cell Signaling Technology [CST]), phospho p44/42 (pERK1/2, 1:2000, #9101S, CST), total JNK (JNK1/2, 1:1000, #9258S, CST), phospho JNK (Thr183/Tyr185, 1:1000, #4668S, CST), total AKT (1:1000, #4691S, CST), phospho AKT (1:1000, Ser473, #9271S, CST) or GAPDH (1:2000,# MAB374, Millipore) overnight at 4°C. Proteins were visualized with chemiluminescence on SuperSignal West Pico Chemiluminiscent Substrate imager (Thermo-Fischer, Massachusetts, USA) after 1h of incubation with appropriate horseradish peroxidase-linked secondary antibody (Sigma-Aldrich). Immunoblots were quantified using ImageJ (image analyzer camera LAS4000, Fujifilm, Tokyo, Japan). Blots were normalized to total GAPDH and an internal standard (IS) was used for normalization between membranes.

### 2.5. Microarray analysis and data pre-processing

Time-resolved gene expression data of stimulated PC12 were recorded at *t* = [1, 2, 3, 4, 5, 6, 8, 12, 24] h and *t* = [1, 2, 3, 4, 6, 8, 12, 24] h for NGF and EGF stimulation, respectively. Control timepoints were measured at 0, 2, 4, 6, 8, 12, 24 h. Total RNA was isolated, labeled and hybridized to an Illumina RatRef-12 BeadChip (Illumina, San Diego, CA, USA) according to the manufacturers protocol. Raw microarray data were processed and quantile normalized using the Bioconductor R package beadarray (Ritchie et al., 2011). Illumina Probes were mapped to reannotated Entrez IDs using the Illumina Ratv1 annotation data (v. 1.26) from Bioconductor. If several probes mapped to the same Entrez ID, the one having the largest interquartile range was retained. This resulted in 15,348 annotated genes, whose expression was further batch corrected according to their chip identity (Johnson et al., 2007). Finally, gene expression time series were smoothed by a 5*th* order polynomial to take advantage of the high sampling rate and replicates at 0, 12, and 24 h. Microarray data have been deposited at Gene Expression Omnibus (GEO) under the accession number GSE74327.

### 2.6. Multi-dimensional scaling

To determine significantly regulated genes over time we performed a multi-dimensional scaling (MDS) using the HiT-MDS algorithm (Strickert et al., 2005). The algorithm projects the 15348 × 15348 distance matrix *D* of the pairwise Euclidean distances between all genes onto a two dimensional space, while preserving distances in *D* as best as possible. Genes varying strongly and uniquely over time will appear as outliers in the MDS point distribution. The uniqueness of a gene expression profile was quantified by fitting a two-dimensional skewed Gaussian distribution (Azzalini, 2015) to the MDS point density function.

### 2.7. Clustering gene expression patterns

To cluster the gene timeseries, we applied the Cluster Affinity Search Technique (CAST), which considers the genes and their similarity over times as nodes and weighted edges of graph, respectively (Ben-Dor et al., 1999). All clusters are considered as unrelated entities and there is no pre-defined number of clusters. Instead a threshold parameter, here *t* = 0.8, determines the affinity between genes and this the final number of gene clusters. Inverse or anti-correlative behavior of genes after NGF or EGF stimulation was determined by fitting a linear model to the smoothed gene expression. Genes having a significant slope with opposite sign and an *r*^2^ > 0.7 were taken as anti-correlated.

### 2.8. Enrichment analysis of transcription factor target gene sets

Upstream analysis for putative transcription factors regulating the EGF and NGF transcriptome responses over time were assessed by a Gene Set Enrichment analysis (Luo et al., 2009) using paired control to treatment samples for each timepoint with an overall cutoff *q*-value < 0.01. As gene sets we used the transcription factor target lists from the Molecular Signatures Database (MSigDB, version 5.0) (Subramanian et al., 2005), for which we mapped the human genes to the rat orthologs using BiomaRt (Huang et al., 2014).

### 2.9. Boolean model

We used a Boolean model framework for dynamic analysis of PC12 cell differentiation. Based on our microarray data and literature knowledge we constructed a highly connected prior knowledge network (PKN) consisting of 63 nodes and 109 edges (cf. Supplementary Table 2). The R/Bioconductor package CellNetOptimizer (CNO) (Saez-Rodriguez et al., 2009) was used to optimize the PKN by reducing redundant nodes, unobservable states and edges. For this we rescaled the qRT-PCR fold change values between 0 and 1 and then transformed with a Hill function $f\left(x\right)=\frac{x^{n}}{x^{n}+k^{n}}$ as suggested in Saez-Rodriguez et al. (2009), where *n* = 2 and *k* = 0.5 denote the Hill coefficient and the threshold, above which a node is considered “on,” respectively. Changing the Hill coefficient between 1 ≤ *n* ≤ 6 did not change the results qualitatively. Model topology optimization was performed via the CellNORdt, which allows fitting with time course data. (See Supplementary Table 3 for stimulus, inhibition and time course data). We set the maximal CPU run time for the underlying genetic algorithm (GA) to 100 s and the relative tolerance to 0.01, using default parameters from the CNO otherwise. A representative evolution of the average and best residual error in a GA run is depicted in Supplementary Image 1A. The solutions quickly converge to a quasi steady state within the time window of simulation of 100 s. The following edges were fixed to prior to optimization based on literature knowledge: NGF → PI3K, NGF → RAS, NGF → PLC, AP1 → NPY, MEK/ERK & JNK → *Jund*, MEK/ERK & JNK → *Junb, Fosl1* & *Jund*→*AP1, Mmp10* → RAS, RAS → MEK, PLC → MEK. Model optimization was performed 100 times and edges were retained, if they appeared in 70% of the runs. This cutoff was chosen to generate a sparse network with robust edges. Performing more runs did not change the results qualitatively (cf. Supplementary Image 1B). Model simulations were performed using the R/Bioconductor package BoolNet (Müssel et al., 2010). The reference publications from which the interactions have been inferred as well as their Boolean transition functions are listed in Supplementary Table 4.

## 3. Results

### 3.1. Gene response of PC12 cells diverges for NGF and EGF on long time scales

To elucidate the dynamic gene response of NGF and EGF, we measured the transcriptome dynamics using Illumina RatRef-12 Expression BeadChips. PC12 cells were either stimulated with NGF or EGF, and collected at the following timepoints: 1, 2, 3, 4, 5, 6, 8, 12, and 24 h. The unstimulated control samples (ctrl) were collected in parallel. Gene expression time series were smoothed by a 5*th* order polynomial to take advantage of the high sampling rate. Finally, we mapped array probes to their respective Entrez IDs, resulting in 15,348 annotated genes.

**Figure 1:**
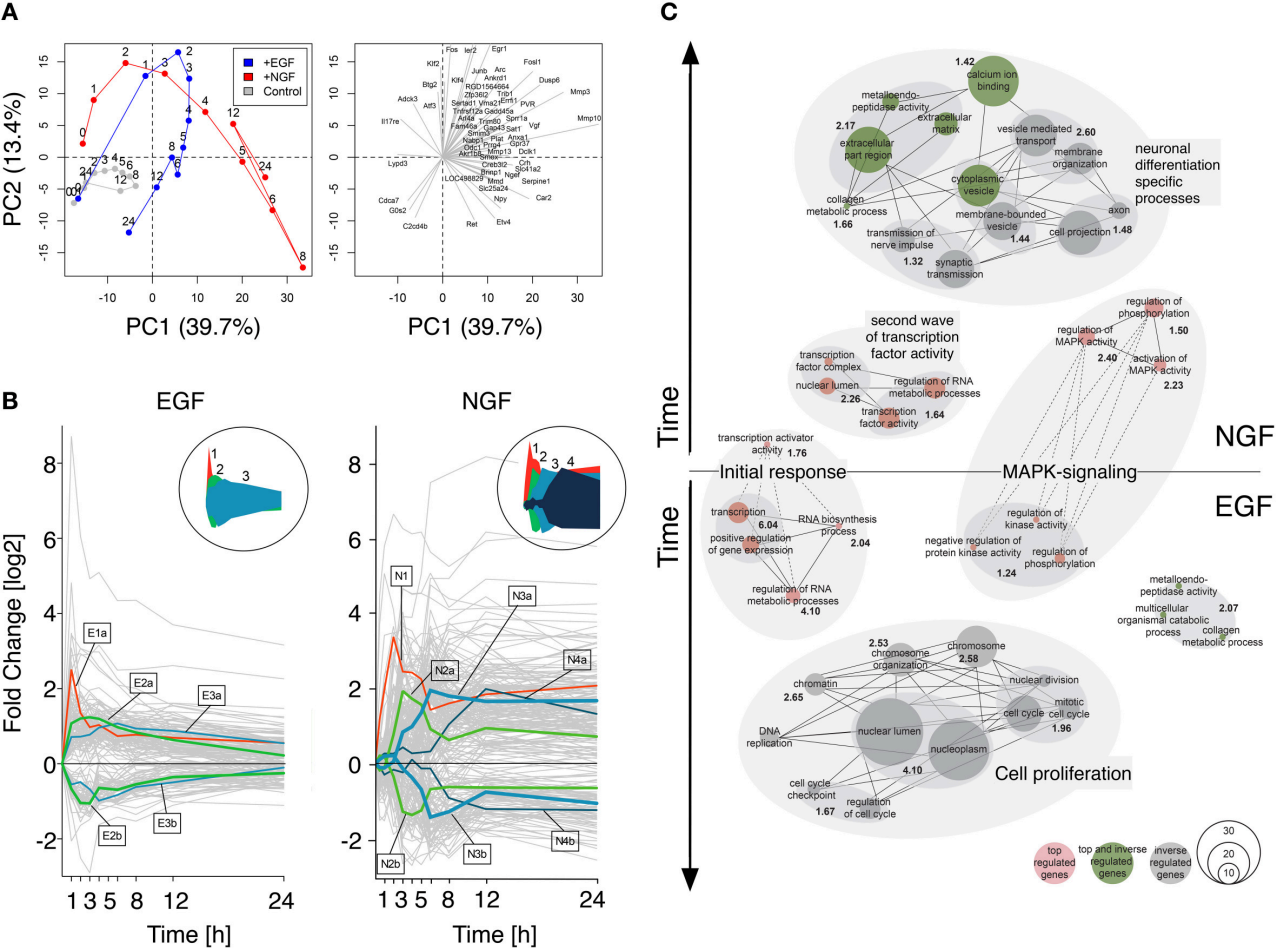
**Gene response dynamics after NGF or EGF stimulation**. **(A)** Principal component analysis (PCA) of the PC12 cell transcriptomes after NGF (red), EGF (blue) and control treatment (gray). The PCA scores (left panel) and loadings (right panel) correspond to the samples and genes, respectively. Samples in the left panel have been connected to guide the eye. Clearly, EGF and NGF samples remain close in the first 3 h and separate at later timepoints, indicating a different cellular phenotype. Right panel: 50 largest loading vectors indicating the impact and time of action of individual genes. Immediate early genes, like *Fos* or *Ier2* point toward early timepoints, while loadings pointing toward the right, like *Vgf* or *Npy*, correspond to late timepoints and are most likely involved in differentiation. **(B)** Expression clusters of top regulated genes. The left and right panels depict the response of individual genes to EGF and NGF stimulation, respectively (gray lines). Cluster centroids are marked by lines with the cluster size encoded by line thickness. The circular inserts depict the cluster centroid envelopes for EGF and NGF, respectively. **(C)** Network representation of functional enrichment of NGF and EGF response genes. The network is comprised of GO-term clusters having a significant enrichment (−*log*_10_(*p*-value) >1.3) as shown in bold black numbers. Red, gray and green nodes contain in this order top-regulated genes, inversely-regulated genes between EGF and NGF or both. The vertical node location corresponds to the peak regulation of their genes, while node size is proportional to the number of genes in a functional category. Edges indicate a gene overlap of >20% between nodes, being drawn as dashed lines, if they are shared between EGF and NGF.

A bi plot of the principal component analysis (PCA) for the 1000 most varying genes depicted a clear separation of the control, NGF and EGF samples. The PCA scores, representing the NGF and EGF treated samples, showed a qualitatively similar behavior up to 4 h after stimulation, yet differed markedly beyond that time (Figure 1A, left). The absolute length and direction of the PCA loadings (Figure 1A, right) indicate the contribution of individual genes to the position of the scores. Correspondingly, several immediate early genes, such as *Junb* (Jun B Proto-Oncogene), *Fos* (FBJ Murine Osteosarcoma Viral Oncogene Homolog), *Ier2* (Immediate Early Response 2), and *Egr1* (Early Growth Response 1) contributed to the early gene response under both EGF and NGF stimulation, while members of the uPAR/Integrin signaling complex, such as *Mmp13/10/3* (Matrix Metallopeptidase 13/10/3), *Plat* (Plasminogen Activator, Tissue) and *Serpine1* (Serpin Peptidase Inhibitor, Clade E, Member 1) determined, among others, the separation of the NGF from the EGF trajectory. Loadings that point toward the control and late EGF response samples, like *Cdca7* (Cell Division Cycle Associated 7) and *G0s2* (G0/G1 Switch 2), are clearly related to cell cycle progression and additionally highlight the difference in proliferation vs. differentiation. In conclusion, the NGF gene response, and thus PC12 cell differentiation, must be determined by late transcriptional feedback events, that trigger and sustain MAPK/ERK signaling.

Next, we sought to functionally analyze the transcriptional differences in early and late gene regulation after EGF and NGF stimulation. For this we selected genes that are (i) strongly regulated (*log*_2_ fold change of < −1.7 or >1.7 in two consecutive timepoints) and (ii) have a unique temporal expression profile according to a multi-dimensional scaling (MDS) analysis (*p*-value < 0.01) (cf. Supplementary Image 2). We found 152 and 402 genes, meeting both criteria, in the EGF and NGF data, respectively, among which 126 genes are shared by both conditions. Figure 1B depicts a clustering of these differentially i.e., top-regulated genes. A cluster affinity search technique (Ben-Dor et al., 1999) identified five EGF (E1-E3b) and seven NGF (N1-N4B) gene response clusters (cf. Supplementary Table 5 and Supplementary Image 3). Interestingly, the EGF stimulus induced a short pulse-like response with rapid return to original gene expression levels, while the NGF stimulus induced a combination of short-impulse like (N1 - N2b) and long sustained gene expression patterns with several clusters (N3a-N4b) sustaining their expression over time (cf. circled insets in Figure 1B).

Figure 1C depicts a network representation of the enrichment analysis using a hypergeometric test on Gene Ontologies (GO). Enriched upregulated biological functions were identified in gene lists E1, E2a, N1, N2a, N3a, N4a and in both groups of inversely regulated genes (cf. Supplementary Table 6). Nodes correspond to GO terms, with numbers indicating the joint enrichment scores. Nodes sharing at least 20 percent of their genes are connected by solid or dotted edges, if the connected nodes lie within a stimulus or across NGF and EGF treatment. Early transcription factor activity is common to both, NGF and EGF signaling, (clusters E1 and N1) as well as MAPK signaling genes (clusters E2a and N3a). The latter, however, is more prominent and enriched at later points in time after NGF stimulation (N3a) compared to the EGF induced response (E2a). Here, a less and earlier enrichment of MAPK signaling genes was seen. Moreover, a second network of transcription factor activity could be identified after NGF stimulation (cluster N2a) that does not have any equivalent after EGF stimulation. It seems, that the initial response (first hour) is controlled by a shared set of top-regulated genes (cf. Figure 1C, dashed lines). The cell-fate specific processes, however, seem to be orchestrated by different set of genes (cf. Figure 1C, separate networks). Many of the genes executing proliferation or differentiation specific processes fall into the category of inversely regulated genes and are not amongst the set of top-regulated genes identified earlier (cf. Figure 1C, green and gray nodes, cf. Material and Methods, cf. Supplementary Table 7). The genes involved in the procession of extracellular matrix and cytoplasmic vesicles, however, constitute an exception: these genes are both top and inverse-regulated (cf. Figure 1C, green nodes).

In summary, functional analysis of the gene clusters revealed an initiation of the differentiation and proliferation process by a shared set of differentially regulated genes. Specific functions, such as transmission of nerve impulse or DNA replication, however, seemed to be executed by two distinct gene groups that are when comparing the EGF to the NGF stimulus inversely regulated over time. Additionally, a second network of genes involved in transcription factor activity was identified in the NGF data set, which lacked a corresponding network in the EGF data set.

### 3.2. Simulation of a Boolean network

Based on the above gene response analyses we sought to identify the mechanisms that sustain MAPK signaling activity after NGF stimulation. Our transcriptome timeseries analysis revealed that the decision process between proliferation and differentiation was spread out over several hours during which transcriptional feedback through an additional set of transcription factors was present after NGF stimulation, only (cf. Figure 1C). To further elucidate the transcription factors upstream of the gene response after EGF or NGF stimulation we performed a gene set enrichment analysis (GSEA) (Luo et al., 2009) on the paired NGF to control and EGF to control transcriptome timeseries. As gene sets we used the motif gene sets from the Molecular Signatures Database (MSigDB v5.0) (Subramanian et al., 2005) and mapped the human genes onto the rat orthologs using BiomaRt (Huang et al., 2014).

**Figure 2:**
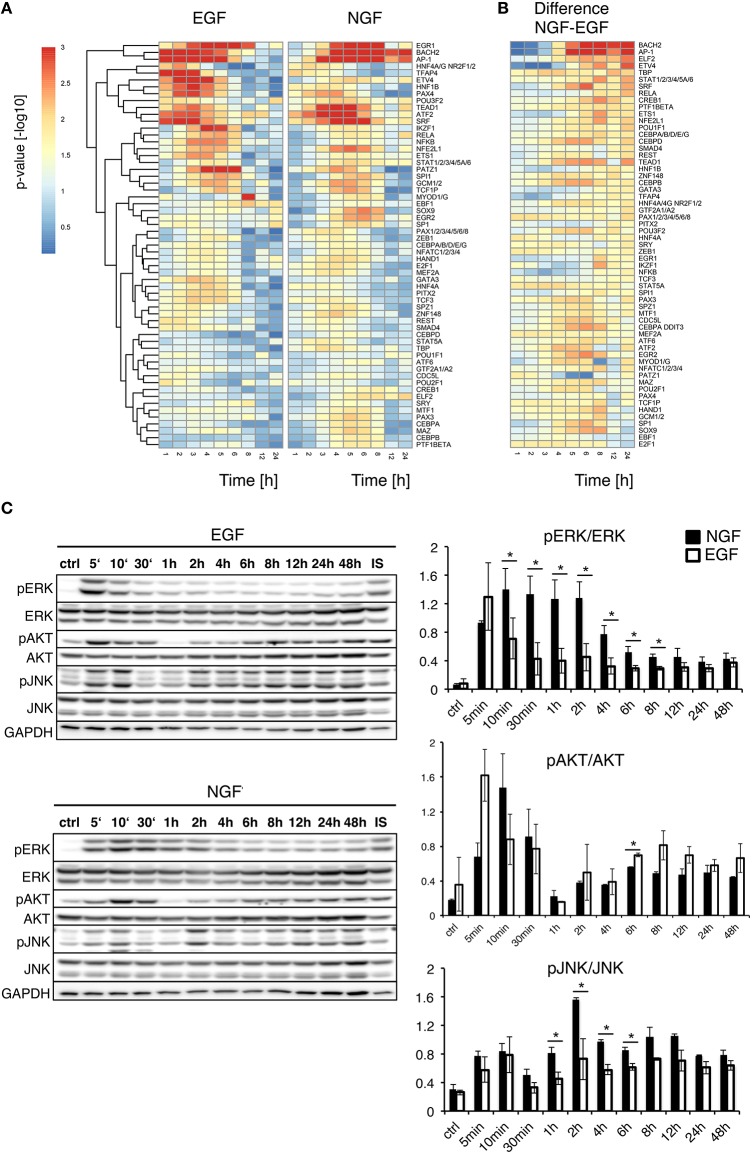
**Upstream analysis of gene expression timeseries**. **(A)** Upstream Gene Set Enrichment Analysis for transcription factors. The heatmaps depict the significance of transcription factors putatively controlling the gene response after EGF (left) or NGF stimulation (right). All TFs are significantly regulated (FDR corrected *p*-value < 0.01) after NGF treatment. TFs have been clustered by their Euclidean distance across all conditions using a complete linkage method. **(B)** Difference in TF *p*-value significance (NGF-EGF). Rows were ordered from the most positive to the most negative difference at *t* = 12 and 24 h. **(C)** Time-resolved quantification of pERK, pAKT and pJNK after EGF and NGF treatment. Original western blots from PC12 cells treated with 75 ng/ml EGF and 50 ng/ml NGF over time. GAPDH is shown as loading control, IS: Internal Standard. Statistical analysis of the pERK/ERK, pAKT/AKT and pJNK/JNK levels are shown on the right panel. An increased and significant higher pERK/ERK level is shown in NGF stimulated (shown as black bars) cells compared to EGF (shown as white bars). A similar trend is visible for pJNK/JNK. A ^*^ denotes a *p*-value < 0.05, data points obtained in duplicates and triplicates.

Figure 2A compares the temporal significance of transcription factors for EGF and NGF stimulation. EGF elicited an early, yet transient significance of all transcription factors, while the time-resolved transcription factor significances for NGF showed early, transient and late activity. Figure 2B depicts the differences in TF significance between NGF and EGF. The most down-regulated TFs relative to EGF are E2F1, EBF1, SOX9 and SP1, all of which are linked to cell proliferation (Bastide et al., 2007; Hallstrom et al., 2008; Györy et al., 2012; Zhang et al., 2014).

Mullenbrock et al. (2011) showed late NGF-induced genes up to 4 h were preferentially regulated by AP1 and CREB (cAMP response element-binding protein). While AP1 was among the most persistently up-regulated transcription factors, we found a transient significance for CREB1, only, peaking at 3 and 6 h, under EGF or NGF stimulation, respectively, which indicated the importance of further TFs beyond that time window. In fact, we found the highest positive differences in the transcription factors BACH2, AP1, as well as ELF2 and ETV4. The latter two belong to the ETS transcription factor family. In particular ETV4, a member of the PEA3 subfamily of ETS, has been shown to promote neurite outgrowth (Fontanet et al., 2013; Kandemir et al., 2014). BACH2, member of the BTB-basic region leucine zipper transcription factor family, is known to down-regulate proliferation and is involved in neuronal differentiation of neoblastoma cells via p21 expression (Shim et al., 2006) and it interacts with the transcription factor MAFF (V-Maf Avian Musculoaponeurotic Fibrosarcoma Oncogene Homolog F) (Kannan et al., 2012) that is necessary for differentiation.

To analyze the early cellular response upon treatment, we additionally compared the phosphorylation levels of pERK, pAKT and pJNK under NGF and EGF stimulation over time (Figure 2C). As expected, pERK increased after NGF and EGF stimulation, showing a persistent up-regulation for 8 h or pulse-like response, respectively. pJNK was continuously up-regulated under NGF relative to EGF stimulation, whereas pAKT responded similar to both stimuli, yet showed a consistently higher phosphorylation under EGF beyond 2 h. Taken together, this corroborates the roles of both pERK and pJNK as well as pAKT in PC12 cell differentiation and proliferation, respectively (Waetzig and Herdegen, 2003; Chen et al., 2012).

Based on the combined transcriptome, upstream transcription factor and protein analyses we next developed a comprehensive prior knowledge interaction network (PKN) for NGF induced PC12 cell differentiation. The PKN comprises key players of known pathways involved in PC12 cell differentiation, such as ERK/PLC/PI3K/JNK/P38/uPAR/NPY and integrin signaling, as well as “linker nodes” to obtain a minimal, yet fully connected network, consisting of 63 nodes and 109 reactions (cf. Supplementary Table 4 for reference publications). The network is depicted in Supplementary Image 4 with differentially regulated genes obtained from our timeseries marked in red and points of inhibition indicated by orange. A Cytoscape readable network format is provided in Supplementary Table 2. Albeit the included PKN pathways are much more complex, our focus was on simulating a biologically plausible signaling flow, including protein signaling, gene response and autocrine signaling as follows: stimulated TrkA receptor activates the downstream pathways PLC/PKC, MAPK/ERK, PI3K/AKT, and JNK/P38. Phosphorylated ERK, PI3K and P38/JNK together activate different transcription factors such as *Fosl1, Fos, Junb, Btg2, Klf2/5/6/10, Cited2, Maff*, and *Egr1*, which are important for PC12 cell differentiation according to our analysis and literature (Cao et al., 1990; Ito et al., 1990; Levkovitz and Baraban, 2002; Gil et al., 2004; Eriksson et al., 2007).

*Junb* and *Fos* initiate the AP1 system, which in turn induces uPA/uPAR signaling, triggering the formation of plasmin (Avraham and Yarden, 2011). The latter is a major factor for the induction of *Mmp3/Mmp10*, linking degradation of the extracellular matrix (ECM) with integrin signaling. The integrins transmit extracellular signaling back via the focal adhesion kinase (FAK) (Singh et al., 2012). FAK activates again the SHC protein, which closes the autocrine signaling. Previous studies reported that uPAR expression is necessary for NGF-induced PC12 cell differentiation (Farias-Eisner et al., 2000; Mullenbrock et al., 2011). A second autocrine signaling loop in the initial PKN putatively acts via the AP1 system, which in turn activates the Neuropeptide Y (NPY/NPYY1 pathway). NPY is a sympathetic co-transmitter that acts via G protein-coupled receptors through interactions with its NPYY1 receptors (Selbie and Hill, 1998; Pons et al., 2008). NPYY1 receptor further activates Ca^2+^ dependent PKC /PLCgamma and subsequently convergences to ERK signaling.

**Figure 3:**
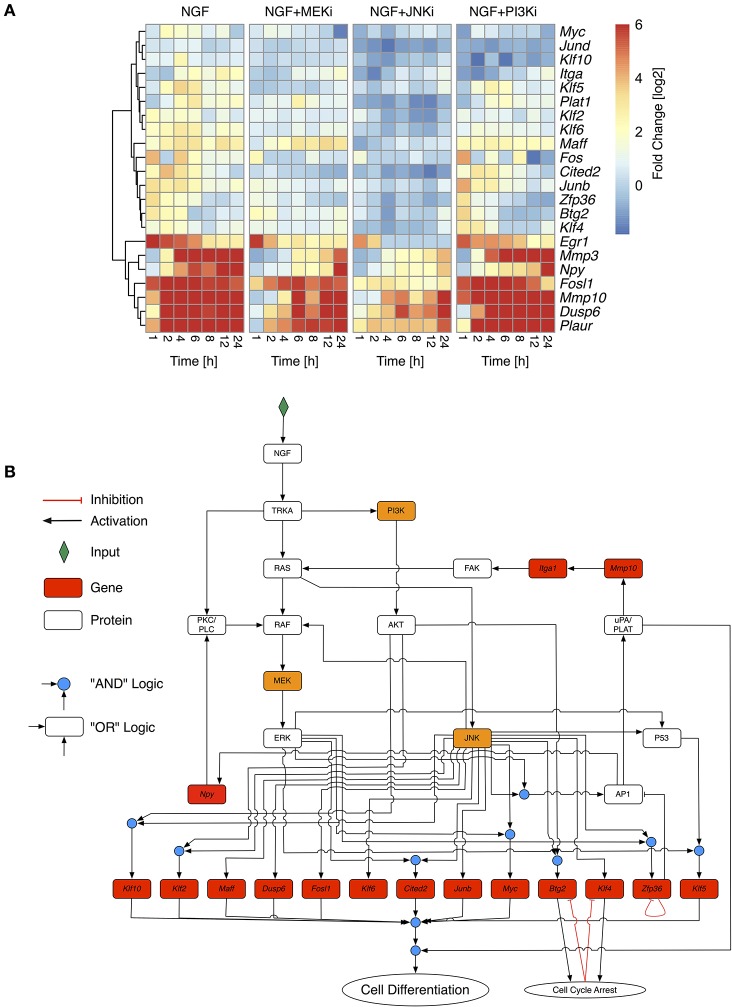
**Selective inhibition of NGF-induced PC12 differentiation**. **(A)** Fold change values of selected response genes in PC12 cells after NGF stimulation under additional inhibition of MEK (NGF+MEKi), JNK (NGF+JNKi), or PI3K (NGF+PI3Ki). Fold change values have been calculated from biological triplicates relative to the unstimulated control per timepoint. To retain the contrast of less variable genes the maximal fold change has been restrained to +6. Genes have been clustered by their Euclidean distance across all conditions using a complete linkage method. **(B)** Optimized Boolean Network based on the training data in **(A)**. Nodes in red have been measured on the transcript level. Orange nodes indicate inhibited proteins.

To optimize the highly connected PKN we used CellNetOptimizer (CNO) (Saez-Rodriguez et al., 2009). The CNO first compresses the network, i.e., it deletes unobservable nodes and then optimizes the network topology using a genetic algorithm. We trained the PKN using gene expression of selected differentially regulated genes under NGF stimulus and inhibition of either MEK, JNK, or PI3K (Figure 3A, MEKi, JNKi and PI3Ki). The overall gene response showed a gradual decline in fold change from NGF via MEK to JNK inhibition, while inhibition of PI3K only moderately impacted the gene expression (Figure 3A). The most affected genes under MEK and JNK inhibition were members of the uPAR signaling pathway, *Mmp10, Mmp3*, and *Plaur* as well as the transcription factors *Fosl1* and *Egr1, Plaur, Dusp6* (Dual Specificity Phosphatase 6) and lastly *Npy*.

Topology optimization using the above perturbations led to a greatly reduced network. Optimization lumped linear pathways into one node, such as the autocrine feedback via uPA/PLAT to *Itga1* and FAK or MEK to ERK transition. The reduced network revealed both MAPK/ERK and JNK as the central network hubs, distributing the upstream signals to downstream genes. It includes two positive feedback via AP1 and uPAR signaling back to FAK and MAPK as well as AP1 to Npy and PKC/PLC back to MAPK. To comply with prior knowledge, we re-expanded linear pathways and added known down-stream target genes, such that the final network, shown in Figure 3B, comprised 32 nodes and 52 edges. We assumed that PC12 differentiation occurs, if the majority of these genes is activated together with uPAR signaling. Due to the inherent difficulty of Boolean networks to incorporate negative feedback loops, we revised the network topology of the reduced network to include transient gene activity of several moderately responding genes. *Klf4* and *Btg2* have been previously been indicated as immediate early genes in PC12 cell differentiation (Dijkmans et al., 2009) and are involved in growth arrest (Tirone, 2001; Yoon et al., 2003), which is a necessary prerequisite for differentiation and degradation of mRNA, respectively. While the explicit mechanism of how *Klf4* and *Btg2* are regulated remains unclear, we assumed an auto-inhibition once they mediated their growth arrest effect. *Zfp36* belongs to the TTP (Tristetraprolin) family of proteins and has been shown to degrade AU-rich mRNAs, particularly of early response genes (Amit et al., 2007). It negatively regulates its own expression (Tiedje et al., 2012) and therefore in the model effectively delays the activity of AP1 before switching itself off. Of note, another member of the TTP protein family, Zfp36l2 (zinc finger protein 36, C3H type-like 2) is constitutively expressed at long times after NGF stimulation (data not shown) and might act as another long-term negative feedback regulator and causing downregulation of *Egr1, Fos*, and *Junb*. Indeed, our experimental data revealed a reduction on gene expression of *Egr1, Fos* and *Junb* over time (Figure 3A).

**Figure 4:**
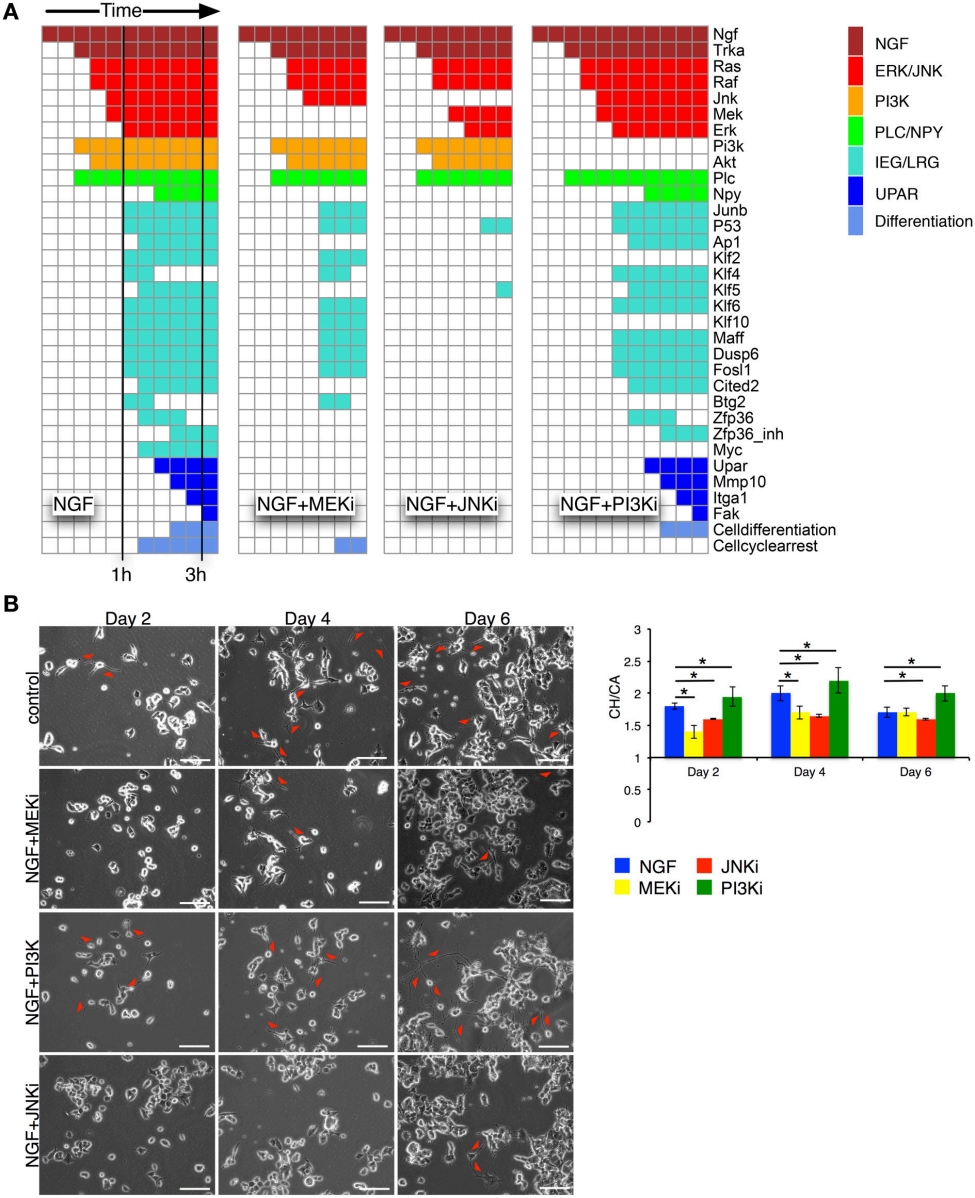
**Network simulation of time sequential pathway activation and experimental validation**. **(A)** The heatmaps depict the path to attractor upon NGF stimulation. Columns correspond to synchronous update steps of the Boolean network. Time progresses from left to right until a steady state is reached. Initially all nodes, except NGF, are set to zero. Colored boxes correspond to activated nodes with the color denoting individual pathways/node categories. Cells are predicted to differentiate, if the node “Cell differentiation” is active, as in the case for NGF, or NGF+PI3Ki treatment. **(B)** Left: phase contrast images for days 2, 4, and 6 are shown for the 4 different conditions: NGF (control), NGF+MEKi, NGF+PI3Ki and NGF+JNKi. Red arrows depict sites of neurite outgrowth in differentiating PC12 cells. Bar: 100 μm. Right: statistical analysis of PC12 cell differentiation from phase contrast imaging for the different conditions are shown as convex hull (CH) to cell area (CA) ratio. Bars show Mean ± SEM, *n* = 2, (^*^t-test *p*-value < 0.05).

We simulated the optimized and re-expanded Boolean network (cf. Supplementary Table 8) using the BoolNet R/Bioconductor package (Müssel et al., 2010), performing two types of simulations. First, we tested the robustness and alternative attractors by setting NGF to “on” and randomly initializing all other network nodes. The nodes were then synchronously updated until a steady state was reached. Within *n* = 10^7^ different simulations, the same final network state with “cell differentiation” set to “on” was always reached. Although this was not an exhaustive search given the number of possible initial network states, it still demonstrated the robustness of the network output. Next, to show the information flow from the NGF receptor to the downstream nodes under different inhibitory conditions, we initialized all nodes except NGF to “off” and performed synchronous updates until a steady state was reached (Figure 4A). Without inhibition, NGF sequentially switches on MAPK, AKT and JNK pathways as well as uPAR signaling. *Klf4, Btg2*, and *Zfp36* become transiently active, with the latter delaying AP1 activity. Blocking MEK (NGF+MEKi) inhibited ERK and thus several downstream targets, including the uPAR feedback. As the latter is assumed indispensable for PC12 cell differentiation, (Farias-Eisner et al., 2000, 2001), the model predicted inhibition of PC12 cell differentiation. The same phenotype is found, when blocking JNK (NGF+JNKi). In comparison to NGF+MEKi it even abrogated the activity of downstream targets altogether. Inhibition of PI3K (NGF+PI3Ki) solely affected PI3K and its downstream target protein AKT and target genes *Maff* and *Klf10*, yet cell differentiation persisted.

Taken together, we developed a core network from the downstream interactome of PC12 cell pathways involved in differentiation. The model captured the dynamic pathway activation after NGF stimulation and various inhibitions. It assigned central and synergistic roles for ERK and JNK in PC12 differentiation with JNK having the largest impact on the network activity.

### 3.3. Model analysis and experimental confirmation

**Figure 5:**
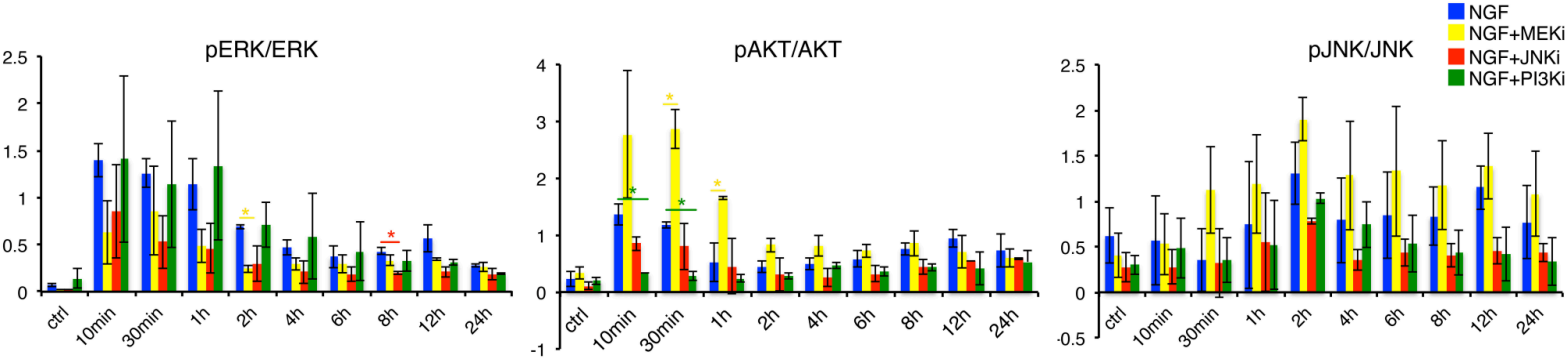
**Quantification of pERK, pAKT and pJNK levels under NGF and individual inhibitor treatments**. Determination of pERK/ERK, pAKT/AKT and pJNK/JNK under NGF, NGF+MEKi, NGF+JNKi and NGF+PI3Ki treatment. Left panel: pERK/ERK levels decrease over time under NGF plus MEK and JNK inhibition. In contrast, PI3K inhibition shows a similar increase and sustained pERK/ERK levels over time compared to NGF treated PC12 cells alone. Interestingly, pAKT/AKT is increased under NGF+MEKi treatment, which is particularly significant in the early timepoints (30 min and 1 h) compared to NGF alone or the other two inhibitors. The latter two show decreased pAKT/AKT levels over time (middle panel). A ^*^ denotes a *p*-value < 0.05, data points obtained in duplicates and triplicates.

Network simulations were confirmed by live phase-contrast imaging (Figure 4B) and western blot analyses (Figure 5). We measured the convex hull (CH) to cell area (CA) ratio of PC12 cells on days 2, 4, and 6. A large convex hull due to extended neurite (marked as red arrow heads in Figure 4B) and small overall cell area is indicative of differentiation (Figure 4B, right panel). Clearly, the continuous CH/CA ratio at day 2 was largest for NGF stimulation and NGF stimulation with additional PI3K inhibition, which corresponded well with the cell differentiation set to “on” in the network simulations under these condition. One can speculate whether inhibition of the pro-proliferative PI3K pathway amplifies cell differentiation, possibly relieving a negative feedback. Indeed, a Western blot of the pERK/ERK ratio depicted a trend to higher ERK phosphorylation relative to NGF stimulation under PI3K inhibition (Figure 5) and phase-contrast images of PC12 cells show more and longer neurites in comparison to cells treated only with NGF or in combination to MEKi and JNKi (Figure 4B, NGF+PI3Ki). Interestingly, image analysis suggested not a stop, but rather a delay of cell differentiation under MEK inhibition. In detail, PC12 cells show no neurites under MEKi after 2 days of combined NGF treatment compared to NGF alone or NGF-PI3Ki. After 4 and 6 days of NGF+MEKi treatment, less cells have neurites in comparison to cells that were only treated with NGF (Figure 4B, NGF+MEKi). In line with literature, pERK levels were reduced, yet pJNK levels were likewise increased, indicating a redirection of protein activity under MEK inhibition (Figure 5, right panel). Likewise, the gene expression showed a reduced, but not completely abolished fold change for *Mmp10* (Figure 3A) and also an up-regulation of Dusp6. Although the discrete Boolean model could not simulate gradual responses, MEK inhibition still resulted in the activation of several downstream target genes necessary for PC12 cell differentiation, while none of these were active under JNK inhibition. In summary, modeling and simulation suggested that PC12 differentiation involved the activity of both JNK/JUN, MAPK/ERK and PI3K/AKT signaling pathways. The establishment of a positive, autocrine feedback loop was indispensable to active late and persistent gene expression.

## 4. Discussion

PC12 cells are a well established model to study the cellular decisions toward proliferation or differentiation. Nevertheless, there is still a lack of understanding on how protein signaling and gene regulation interact on different time scales to decide on a long-term, sustained phenotype. Given the fact that PC12 cell cycle and differentiation last up to 4 and 6 days, respectively (Greene and Tischler, 1976; Luo et al., 1999; Adamski et al., 2007), late events occurring beyond the first hours are most likely to be important for sustaining the cellular decision. However, few studies that have compared the long-term effect of EGF and NGF in PC12 cells. They focused either on NGF alone (Dijkmans et al., 2008, 2009), on individual (Angelastro et al., 2000; Marek et al., 2004; Lee et al., 2005; Chung et al., 2010), or early time-points (Mullenbrock et al., 2011).

Previous studies have identified expression of immediate early genes (IEG), such as *Egr1, Junb*, and *Fos* together with delayed early genes (DEG), like *Dusp6, Mmp3/10, Fosl1*, and *Atf3* as necessary for PC12 cell differentiation (Vician et al., 1997; Levkovitz et al., 2001; Dijkmans et al., 2008; Mullenbrock et al., 2011). However, we found all these genes strongly regulated by both EGF and NGF stimulation (Supplementary Table 5), however, showing differences in their expression kinetics (Figure 1). Akin to differences in the pERK dynamics, these results suggest that cellular decisions toward differentiation or proliferation are driven by the differences in the gene expression kinetics.

It has been suggested before that distinct cellular stimuli activate similar sets of response genes, whose expression dynamics, rather than their composition, determine cellular decisions (Murphy and Blenis, 2006; Amit et al., 2007; Yosef and Regev, 2011). Single expression bursts are likely to stimulate proliferation, while complex, wave-like expression patterns induce differentiation (Bar-Joseph et al., 2012). Accordingly, EGF elicited a pulse-like gene response, while NGF induced a complex, wave-like gene response (Figure 1B). After EGF stimulation the expression of IEGs, *Egr1, Fos*, and *Junb* was quickly attenuated through the rapid up-regulation of their negative regulators, namely *Fosl1, Atf3, Maff*, *Klf2*, and *Zfp36l2* and contributing to a pulse-like gene expression. Furthermore, *Fosl1* counteracts *Fos* and AP1 (Hoffmann et al., 2005) and *Atf3* has been shown to modulate *Egr1* activity (Giraldo et al., 2012), while *Maff* and *Klf2* negatively regulate serum response and STAT-responsive promoter elements (Amit et al., 2007). The same genes respond after NGF stimulation, however with a delayed response and might be one of the reasons for the stronger and longer gene and pERK response under NGF stimulation (Murphy et al., 2002, 2004; Murphy and Blenis, 2006; Saito et al., 2013).

A recent study by Mullenbrock et al. (2011) compared the transcriptome response of PC12 cells to EGF and NGF stimulation up to 4 h. Using chromatin immunoprecipitation they found a preferential regulation of late genes through AP1 and CREB TFs after NGF stimulation, which is in line with our findings (Figure 2A). However, we predicted a constitutive significance for AP1 up to 24 h, while CREB1 displayed a transient importance, being most abundant at 6 h after stimulation. Furthermore, we found a switch in the composition of transcriptional master regulators between 4 and 12 h. During this time, late TFs, such as BACH2, ETS1 and ELF2 become active.

Supplementary Image 5 depicts a Volcano plot of their target genes. Beyond the early gene targets, such as *Fosl1* or *Junb*, the late TFs additionally target related to cytoskeleton, morphogenesis and apoptosis, such as Tumor Necrosis Factor Receptor Superfamily, Member 12A (*Tnfrsf12a*), Doublecortin-Like Kinase 1 (*Dclk1*), Nerve Growth Factor Inducible *Vgf*, Coronin, Actin Binding Protein, 1A (*Coro1a*, Growth Arrest And DNA-Damage-Inducible, Alpha (*Gadd45a*) and *Npy*. Of note, we found *Rasa2* among the targets, which has recently been identified as a driver for differentiation through a negative feedback between PI3K and RAS (Chen et al., 2012).

A recent study by Aoki et al. (2013) investigated the down-stream gene response upon light-induced intermittent and continuous ERK activation in normal rat kidney epithelial cells. Similar to the TF activity after EGF and NGF stimulation in PC12 cells, intermittent pERK activity caused up-regulation of *Fos, Egfr, Ier2*, and *Fgf21*, which were putatively controlled through serum response factor (SRF) and CREB binding sites, while sustained pERK activity caused gene regulation controlled by AP1 and BACH1. One can speculate that it is more the temporal dynamics of pERK and less the upstream ligands, such as EGF or NGF, that eventually encode the transcriptional program deciding on the cell fate.

To elucidate the various pathways and downstream target genes under NGF stimulation we constructed a Boolean model based on our transcriptome and additional literature data. A prior knowledge network revealed a highly interconnected pathway map transmitting NGF-induced signals. Training the network via inhibition of MEK, JNK or PI3K reduced the number of edges and nodes by about 80% and revealed the MAPK/JNK pathway as second signaling hub next to MAPK/ERK. Moreover, blocking the JNK pathway had a more drastic effect on cell differentiation than blocking MAPK/ERK via inhibition of MEK through UO126. Indeed, studies on the effect of MEK inhibition for PC12 cell differentiation are inconclusive. Early studies report how MEK inhibition completely averted PC12 cell differentiation (Pang et al., 1995; Klesse et al., 1999), while recent experiments suggest a decrease, rather than full inhibition of differentiation (Levkovitz et al., 2001; Chung et al., 2014). Our results were in line with the latter. Despite a significant reduction in pERK (Figure 5), our cell morphology measurements detected merely a decrease in the formation of neurites, rather than full inhibition of differentiation. The reason for this discrepancy could lie in the time scale of observation. MEK inhibition delayed differentiation and it took 6 days to eventually overcome this delay (Figure 4B). This confirmed the modeling results, which established JNK as key regulator that is closely interlinked with MAPK/ERK signaling. In concert with pERK, also pJNK becomes constitutively active upon NGF stimulation (Figure 2C). Moreover, blocking pERK through MEK even increased pJNK (and pAKT) levels, while pERK decreased after JNK inhibition, verifying a crosstalk between JNK and ERK pathways. Previous reports suggested such a crosstalk due to dual-phosphatase interaction (Fey et al., 2012), while other studies proposed that JNK phosphorylates RAF (Adler et al., 2005; Chen et al., 2012) and thereby contributing to MAPK/ERK activity. However, the mechanistic details governing the crosstalk remain unclear so far. In conclusion, while previous studies assigned parallel, non-redundant roles to MAPK/ERK and MAPK/JNK (Waetzig and Herdegen, 2003), our results show that JNK signaling might be even the main driver for PC12 cell differentiation.

Next to the negative feedback loops through *Klf4, Zfp36*, and *Btg2*, arresting cell cycle and attenuating mRNA abundance, we included also two positive feedback loops via uPAR and integrin signaling as well as through Neuropeptide Y and PKC/PLC signaling. Positive feedback loops are a common regulatory pattern in molecular biology to induce bistability switch-like behavior, particularly in cell fate decisions and differentiation (Xiong and Ferrell, 2003; Mitrophanov and Groisman, 2008; Kueh et al., 2013). In fact, multiple feedbacks deciding between PC12 cell differentiation and proliferation, have been studied on the level of MAPK signaling (Santos et al., 2007; von Kriegsheim et al., 2009). Recently, Ryu et al. (2015) used a FRET construct to quantify pERK dynamics on a single cell level after growth factor stimulation. While the cell population average still resembled the hitherto described transient and sustained pERK activity after respective EGF and NGF stimulation, the authors found a highly heterogenous response on the single cell level. Pulsed stimulation, however, not only synchronized MAPK activity between cells, but also triggered PC12 differentiation upon EGF stimulation, if the integrated pERK signal was large enough. The authors concluded that thus not only MAPK signaling, but also further pathways are responsible for the cell fate decision. Sparta et al. (2015) used a similar experimental approach to single cell response of human MCF10A-5e cells to show that EGFR activity induced a frequency modulation response, while TrkA activity caused amplitude modulation of pERK levels. The authors explained these finding by additional receptor-dependent signaling networks beyond the core Ras-Raf-MEK-ERK pathway. Extending on this idea, our data and model suggest autocrine signaling as further feedbacks that sustain the expression of differentiation inducing TFs. Indeed, uPAR and also *Npy* activity were strongly correlated with differentiation (Figure 3A) and neither Npy nor uPAR signaling were activated upon EGF stimulation (data not shown). In line with this finding previous studies reported that uPAR expression is necessary for NGF-induced PC12 cell differentiation (Farias-Eisner et al., 2000; Mullenbrock et al., 2011). SERPINE1 regulating the plasminogen activator-plasmin proteolysis was shown to promote neurite outgrowth and phosphorylation of the TrkA receptor and ERK (Soeda et al., 2006, 2008). In our model we included the necessity of uPAR signaling though the activation of late genes, such as *Klf5*, yet the causal relationship between uPAR signaling and late gene expression remains unclear. However, uPAR signaling could constitute the additional positive feedbacks beyond MAPK signaling that were predicted by Ryu et al. (2015), which would be interesting to test on the single cell level. Reporters for uPAR and/or JNK activity should likewise show a heterogenous activity and correlate with the per-cell differentiation status, which could potentially be modeled within a stochastic differential equation framework.

In conclusion, our approach has identified the short and long-term transcriptional activity in PC12 cells after NGF and EGF stimulation. Modeling the pathway orchestration using a Boolean model we identified feedback regulations beyond MAPK signaling that attenuate and sustain the cellular decision toward differentiation. Extending on previous studies we established JNK as a key player in PC12 cell differentiation that might have equal, if not even more importance than ERK during this process. Over time AP1 was accompanied by a variety of transcription factors serving signal attenuation, signal maintenance and morphological change of the cell, which demonstrates that the decision toward differentiation is a time sequential process over at least 12 h.

## Author contributions

MF, SKn, MK, SK, and BO performed the experiments. MF, SKn, AS, and BO performed the data analysis. HB And MB conceived the project, performed the data analysis and wrote the manuscript with BO. All authors approved the final manuscript.

### Conflict of interest statement

The authors declare that the research was conducted in the absence of any commercial or financial relationships that could be construed as a potential conflict of interest.
