# Supplementary material for: Boolean Modeling Reveals the Necessity of Transcriptional Regulation for Bistability in PC12 Cell Differentiation
Source: Front Genet. 2016 Apr 14;7:44. doi: 10.3389/fgene.2016.00044 (PMC4830832; doi:10.3389/fgene.2016.00044)
Supplement: Supplementary file 2 [file Table2.docx]

**Supplementary Table 2**. Simple interaction format file describing the prior knowledge network (PKN). The SIF file contains all interactions of the prior knowledge network and can be visualized by the open source software Cytoscape (http://www.cytoscape.org/) or the R/Bioconductor package CellNetOptimizer (http://http://www.cellnopt.org/).

NGF 1 TrKA

TrKA 1 SHC

SHC 1 GRB2

GRB2 1 SOS

SOS 1 RAS

TrKA 1 FRS2

FRS2 1 C3G

C3G 1 RAP1

RAP1 1 FAK

TrKA 1 PI3K

PI3K 1 AKT

RAS 1 RAF

RAF 1 MEK

MEKK1 1 MEK

MEK 1 ERK

ERK 1 RSK

TrKA 1 PLC

GNAI1 1 PLC

PLC 1 DAG

PLC 1 Ca2+

Ca2+ 1 PKC

DAG 1 PKC

PKC 1 RAF

AP1 1 uPAR

uPAR 1 uPA-PLAT

uPA-PLAT 1 Plasmin

Plasmin 1 Mmp10

Mmp10 1 ECM

ECM 1 Itga1

Itga1 1 FAK

FAK 1 SHC

AP1 1 Npy

Npy 1 NPYY1

NPYY1 1 GNAI1

RAS 1 RAC1

RAC1 1 MEKK4

RAC1 1 MEKK1

MEKK4 1 MKK6

MEKK4 1 Mapk3k

MEKK1 1 MKK7

MEKK1 1 MKK4

MKK7 1 JNK

MKK4 1 JNK

MKK6 1 P38

Mapk3k 1 P38

P38 1 ATF2

ERK 1 ATF2

JNK 1 ATF2

RSK 1 CREB

AKT 1 CREB

MSK1/2 1 CREB

ERK 1 MSK1/2

P38 1 MSK1/2

RSK 1 SRF

Egr1 1 Arc

CREB 1 Arc

ERK 1 Junb

JNK 1 Junb

AKT 1 Junb

ERK 1 Jund

JNK 1 Jund

Fos 1 AP1

Jund 1 AP1

Junb 1 AP1

Fosl1 1 AP1

ERK 1 Klf4

JNK 1 Klf4

AKT 1 Klf4

AKT 1 Klf2

JNK 1 Klf2

ERK 1 Klf2

ERK 1 Klf5

AKT 1 Klf5

P53 1 Klf5

ERK 1 P53

JNK 1 P53

AKT 1 P53

JNK 1 Klf6

P53 1 Klf6

AKT 1 Tieg1

ERK 1 Tieg1

JNK 1 Tieg1

ERK 1 Cited2

P53 1 Cited2

CREB 1 Cited2

ERK 1 Btg2

AKT 1 Btg2

JNK 1 Zfp36

ERK 1 Zfp36

JNK 1 Myc

ERK 1 Myc

AKT 1 Myc

ETS 1 Dusp6

ERK 1 ETS

JNK 1 ETS

ERK 1 Egr1

AKT 1 Egr1

JNK 1 Egr1

ERK 1 Fos

JNK 1 Fos

AKT 1 Fos

ERK 1 Stat3

JNK 1 Stat3

ERK 1 Maff

JNK 1 Maff

ATF2 1 Maff

ERK 1 Fosl1

JNK 1 Fosl1

AKT 1 Fosl1
