# Supplementary material for: Boolean Modeling Reveals the Necessity of Transcriptional Regulation for Bistability in PC12 Cell Differentiation
Source: Front Genet. 2016 Apr 14;7:44. doi: 10.3389/fgene.2016.00044 (PMC4830832; doi:10.3389/fgene.2016.00044)
Supplement: Supplementary file 4 [file Table4.pdf]

Supplementary Table 4. Reactions of prior knowledge network (PKN) and Pubmed IDs of respective publications that report the interactions.

No	Reactions	Pubmed ID
1	NGF→TrkA	7571013
2	TrkA→SHC	7541035
3	FAK→SHC	9032297,10347094
4	SHC→GRB2	7513258, 7486657
5	GRB2→SOS	7739560, 8577724
6	SOS→RAS	9690470, 8828504, 11777939
7	TrkA→FRS2	10629055, 10092678
8	FRS2 →C3G	10092678, 11278445
9	C3G→RAP1	8524240, 8524240, 12734187, 10753869, 17093062, 9856955, 9853756
10	RAS→RAF	18316782, 9069260
11	PKC→RAF	7534287, 8321321
12	RAF →MEK	10082509, 19074159
13	MEKK1→MEK	16006144
14	MEK→ERK	9069255, 18321244, 20665674
15	ERK→RSK	8674434, 11752651, 11591711, 11865188, 11278279
16	TrkA→PI3K	12133829, 1380963
17	PI3K →AKT	9321394, 17826033
18	TrkA→PLC	11283249, 1712104
19	G(i/o)→PLC	18508528
20	PLC →DAG	11048639, 10579907
21	PLC →Ca^{2+}	11048639, 2550825
22	DAG →PKC	2550825, 12495431
23	Ca^{2+} →PKC	3014651
24	AP1→uPAR	15963460
25	uPAR→uPA/*PLAT*	8798468
26	uPA/*PLAT*→Plasmin	1381366, 6891264, 1378833, 10190280, 11359936, 15688295
27	Plasmin→*Mmp3/10*	9398846
28	*Mmp3/10* → ECM	10469644, 8612226
29	ECM →*Itga1*	10215583
30	*Itga1*→*FAK*	16919435, 9616126
31	RAP-1→FAK	23209645, 11809811
32	AP1→*Npy*	11150315
33	Npy→NPYY1	18641693
34	NPYY1→G(i/o)	9089810, 12209475, 118641693, 9263910, 12710520
35	RAS →RAC1	9808620, 10793146, 16203995
36	RAC1→MEKK4	9079650
37	RAC1→MEKK1	11057896, 9305638, 14581471, 9712898
38	MEKK4→MKK6	17726008, 9305639
39	MEKK4→*Mapk3k*	17726008, 1154276
40	MEKK1→MKK7	11416147
41	MKK7→JNK	12624093, 9535930
42	MKK4→JNK	9890951, 11057897
43	*Mapk3k* →*P38*	8622669, 11304531
44	MKK6 →P38	11304531
45	P38→ATF2	7535770, 12110590
46	ERK→ATF2	12110590
47	JNK→ATF2	10777545, 25456131
48	RSK→CREB	8688081, 11909979
49	AKT→CREB	10579998, 12388598, 10753867, 9829964
50	MSK1/2→CREB	11909979

No	Reactions	Pubmed ID
51	ERK→MSK1/2	9687510
52	P38→MSK1/2	9687510
53	RSK→SRF	8413226, 16868029
54	EGR1→ *ARC*	21887136
55	CREB→ *ARC*	19116276
56	ERK→ *JUNB*	22443687
57	JNK→ *JUNB*	22443687, 14617628
58	AKT→ *JUNB*	21135252
59	ERK→ *JUND*	12835716
60	JNK→ *JUND*	14676207, 12226747
61	*FOSL1*→AP1	11150315
62	*JUND*→AP1	15564374, 11150315
63	*FOS*→AP1	9069263, 12226747, 15564374
64	*JUNB*→AP1	9069263, 12226747, 15564374
65	ERK→ *KLF4*	12226747, 20711222, 22307056
66	JNK→ *KLF4*	20711222
66	AKT→ *KLF4*	23762260
68	ERK→ *KLF2*	24905170
69	AKT→ *KLF2*	20525885, 15834135, 16571724
70	JNK → *KLF2*	20032497
71	ERK→ *KLF5*	19628677
72	AKT→ *KLF5*	18424767
73	P53→ *KLF5*	16595680
74	JNK→P53	9732264,9724739
75	ERK→P53	10781582
76	AKT→P53	17126425
77	JNK→*KLF6*	19333010
78	P53→*KLF6*	18174288
79	AKT→ *KLF10*	21873430, 19013137
80	ERK→*KLF10*	117574220, 19013137
81	JNK→*KLF10*	23164821
82	CREB→*CITED2*	11279224, 15051727
83	ERK→*CITED2*	23082118, 17283246
84	P53→*CITED2*	18495890
85	JNK→*BTG2*	11267995
86	AKT→*BTG2*	14734530
87	JNK→*ZFP36*	12646273
88	ERK→*ZFP36*	22433566
89	JNK→MYC	11597764, 10551811, 16483932
90	ERK→MYC	11597764, 16899113, 16483932
91	AKT→MYC	22461507
92	ERK→ETS1	18039929, 15572696, 25294825
93	JNK→ETS1	23966295, 11027273
94	ETS1→*Dusp6*	18321244, 20097731
95	ERK→*Egr1*	12393577, 16858414, 20554967
96	AKT→*Egr1*	14769801, 18844239
97	JNK→*Egr1*	19681663, 22577133
98	ERK→*Fos*	12134156, 16123044, 20493519
99	AKT→*Fos*	23408974, 9690619
100	JNK→*Fos*	14511403, 10972672, 11410534
101	ERK→*Stat3*	9343414, 10521505, 11553624
102	JNK→*Stat3*	10521505, 11553624
103	ERK→*Maff*	21494257, 19251651
104	JNK→*Maff*	19013137
105	ATF2→*Maff*	21278380
106	ERK→*Fosl1*	12197835, 11756554
107	AKT→*Fosl1*	16490785, 17872495
108	JNK→*Fosl1*	12665585
