# Supplementary material for: Boolean Modeling Reveals the Necessity of Transcriptional Regulation for Bistability in PC12 Cell Differentiation
Source: Front Genet. 2016 Apr 14;7:44. doi: 10.3389/fgene.2016.00044 (PMC4830832; doi:10.3389/fgene.2016.00044)
Supplement: Supplementary file 8 [file Table8.pdf]

Supplementary Table 8. Network file for use with the R BoolNet library.
It describes the optimized Boolean network used in this manuscript simulating the NGF-induced PC12 cell differentiation.

```
targets,factors
NGF,NGF
TRKA,NGF
RAS,TRKA|FAK
RAF,(PLC|JNK|RAS)
JNK,RAS
MEK,RAF
ERK,MEK
PI3K,NGF
AKT,PI3K
PLC,NGF|NPY
NPY,AP1
JUNB,JNK
P53,ERK|JNK
AP1,JNK&ERK
KLF2,PI3K&JNK
KLF4,JNK&!CellCycleArrest
KLF5,P53&ERK
KLF6,JNK
KLF10,PI3K&JNK
MAFF,JNK
DUSP6,JNK
FOSL1,JNK
CITED2,ERK&JNK
BTG2,PI3K&JNK&!CellCycleArrest
ZFP36,JNK&ERK&!ZFP36_inh
ZFP36_inh,ZFP36[-2]|ZFP36_inh
MYC,JNK&ERK&AKT
UPAR,AP1
MMP10,UPAR
ITGA1,MMP10
FAK,ITGA1
CellDifferentiation,UPAR&maj(DUSP6,FOSL1,CITED2,KLF5,KLF6,ZFP36,MYC,JUNB,MAFF)
CellCycleArrest,CellCycleArrest|(KLF4&BTG2)
```
