# Supplementary material for: Boolean Modeling Reveals the Necessity of Transcriptional Regulation for Bistability in PC12 Cell Differentiation
Source: Front Genet. 2016 Apr 14;7:44. doi: 10.3389/fgene.2016.00044 (PMC4830832; doi:10.3389/fgene.2016.00044)
Supplement: Supplementary file 9 [file Image1.pdf]

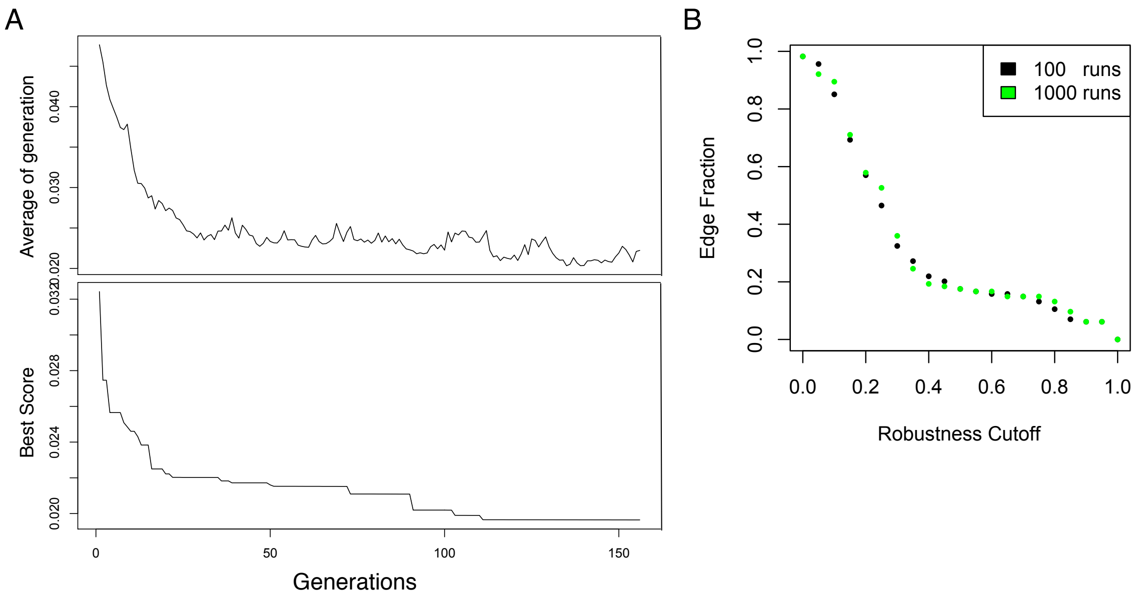

Supplementary Figure 1. Model topology optimization. (A) Evolution of residual error along generations in a genetic algorithm optimization. The plots depict the residual error between the simulated Boolean model and scaled experimental data along > 150 generations of a genetic algorithm. Top: average error across the population of 50 Boolean networks. Bottom: residual error for the best performing network. (B) Fraction of retained edges in the PKN network versus robustness cutoff. The plot depicts the fraction of retained edges in the original prior knowledge network as a function of the relative frequency of finding an edge after network optimization with a genetic algorithm, denoted as robustness cutoff. Note how the fraction of retained edges first drops linearly with the robustness cutoff up to 0.4 and leveling off up to $0.7 - 0.8$ thereafter. Increasing the number of independent GA runs did not change qualitatively the edge fraction behavior. For this reason we chose a robustness cutoff $= 0.7$ for network optimization.
