# Supplementary material for: Boolean Modeling Reveals the Necessity of Transcriptional Regulation for Bistability in PC12 Cell Differentiation
Source: Front Genet. 2016 Apr 14;7:44. doi: 10.3389/fgene.2016.00044 (PMC4830832; doi:10.3389/fgene.2016.00044)
Supplement: Supplementary file 10 [file Image2.pdf]

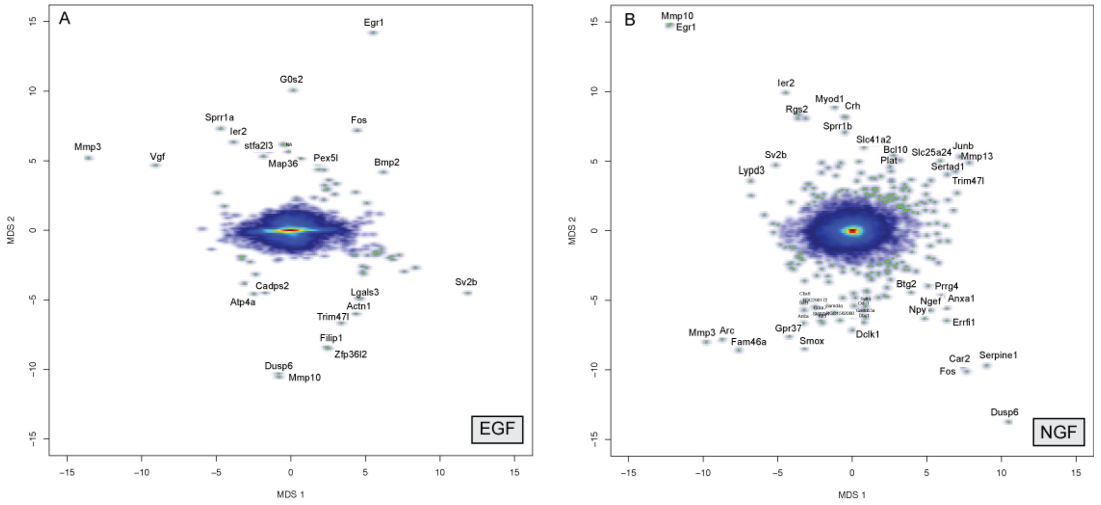

Supplementary Figure 2. Density plot of the multi-dimensional scaling of the transcriptome time-series after EGF and NGF stimulation. The matrix D, containing the pairwise Euclidean distance between all genes, was mapped onto a 2D space. Gene distance in 2D is proportional to their Euclidean distance and therefore the similarity in expression over time. Likewise, genes having a strong and unique response pattern show as outliers in the density plot. (A): transcriptome response of EGF treated cells. (B): transcriptome response of NGF treated cells. The most significantly regulated genes are annotated by their gene symbol.
