# Supplementary figures and images for: Boolean Modeling Reveals the Necessity of Transcriptional Regulation for Bistability in PC12 Cell Differentiation
Source: Front Genet. 2016 Apr 14;7:44. doi: 10.3389/fgene.2016.00044 (PMC4830832; doi:10.3389/fgene.2016.00044)

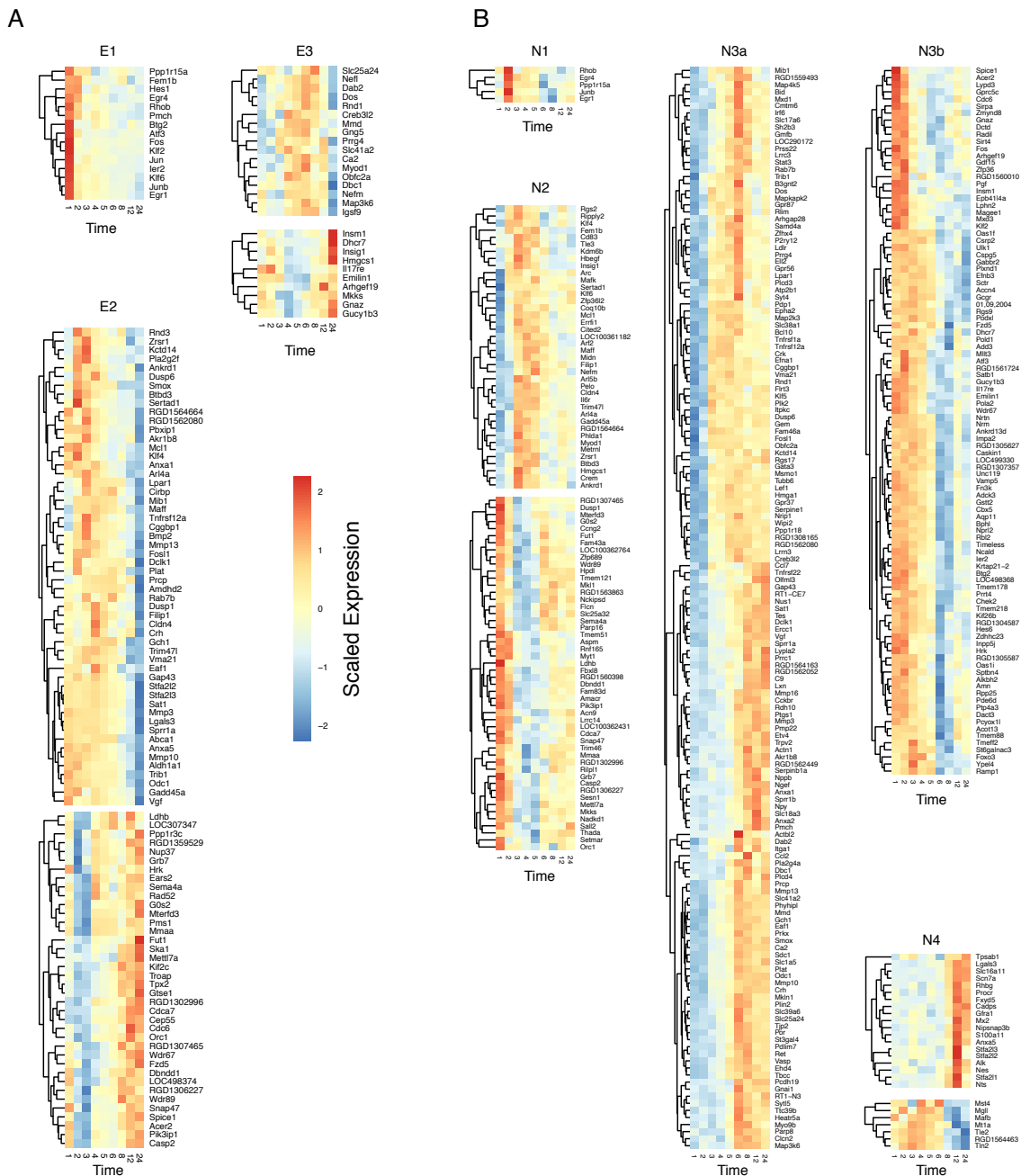

Supplement: Supplementary file 11 [file Image3.pdf]
