# Supplementary material for: Boolean Modeling Reveals the Necessity of Transcriptional Regulation for Bistability in PC12 Cell Differentiation
Source: Front Genet. 2016 Apr 14;7:44. doi: 10.3389/fgene.2016.00044 (PMC4830832; doi:10.3389/fgene.2016.00044)
Supplement: Supplementary file 12 [file Image4.pdf]

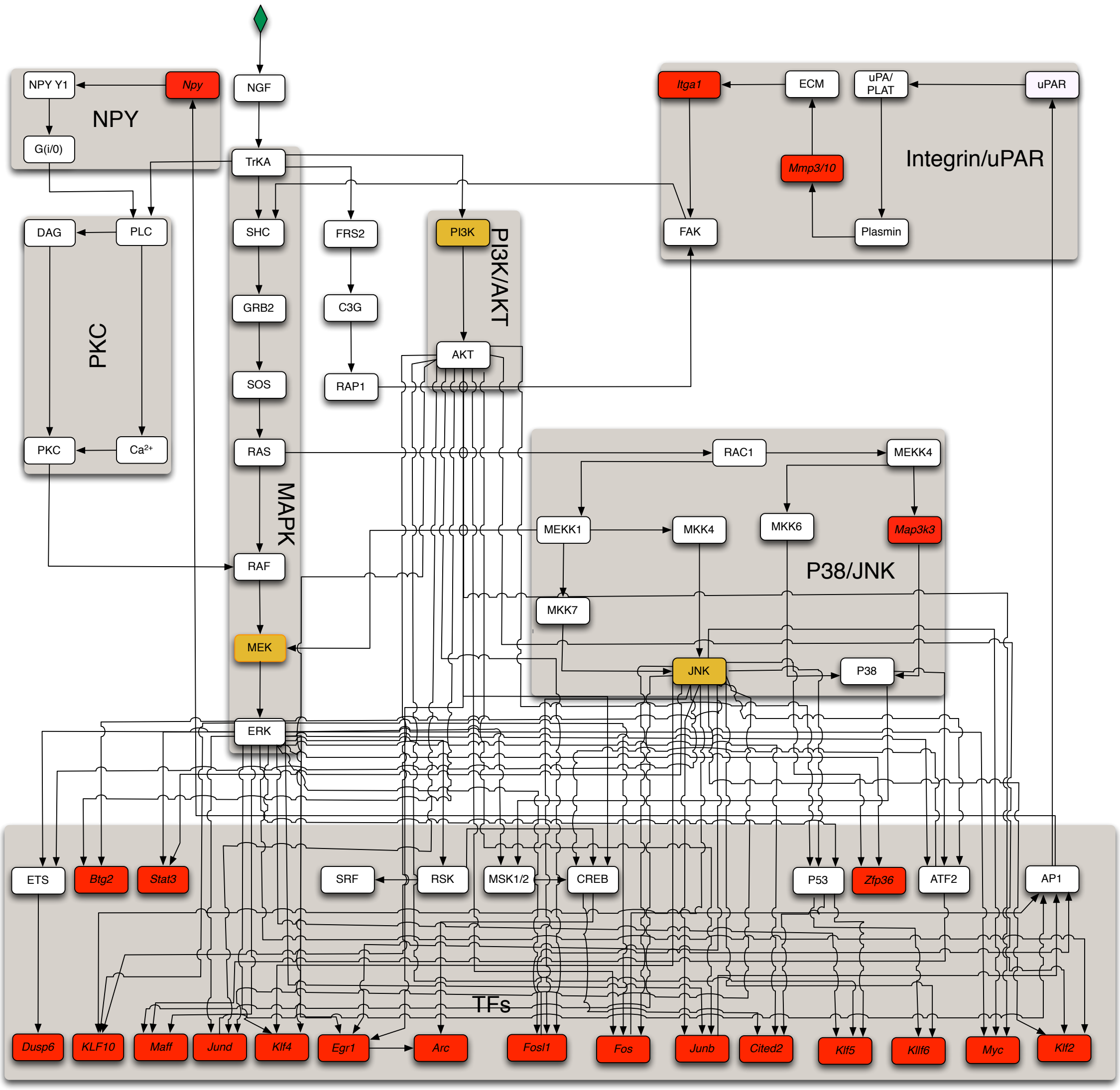

Supplementary Figure 4. Prior Knowledge Network of pathways and gene response involved in PC12 differentiation. The prior knowledge network for PC12 cell differentiation was derived combining both transcriptome responses and literature mining. Orange and red nodes denote points of inhibition and differentially regulated genes found from transcriptome analysis, respectively. The colored backdrops indicate node membership with respect to pathways. The green diamond denotes the input of NGF stimulation.
