# Supplementary material for: Boolean Modeling Reveals the Necessity of Transcriptional Regulation for Bistability in PC12 Cell Differentiation
Source: Front Genet. 2016 Apr 14;7:44. doi: 10.3389/fgene.2016.00044 (PMC4830832; doi:10.3389/fgene.2016.00044)
Supplement: Supplementary file 13 [file Image5.pdf]

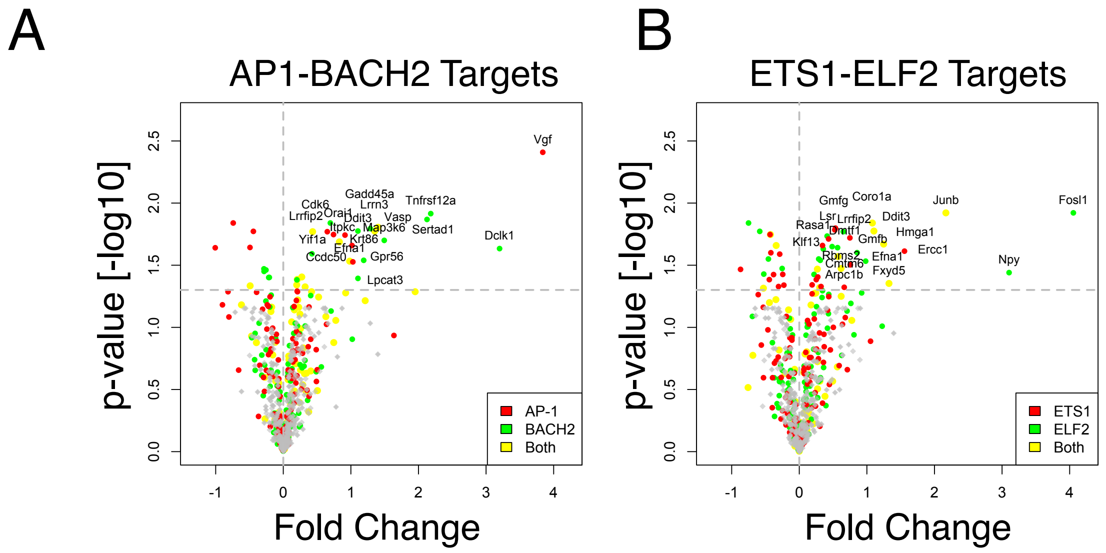

Supplementary Figure 5. Volcano plots of the gene targets for (A) AP1-BACH2 and (B) ETS1-ELF2. The fold change and significance have been calculated by a moderated t-test of the 0h expression versus the gene expression at 12 and 24 hours after stimulation. The colors indicate whether the genes are target by the both (yellow) or the individual transcription factors (red and green). The grey dots depict the sample genes upon EGF stimulation. Significantly up-regulated genes (adj. p-value < 0.05 and fold change > 0.5) are annotated with their gene symbol.
